# Knowledge, awareness and practice of infection control by health care workers in the intensive care units of a tertiary hospital in Nigeria

**DOI:** 10.4314/ahs.v18i1.11

**Published:** 2018-03

**Authors:** Majeed Babajide Adegboye, Suleiman Zakari, Bola Abdulkadir Ahmed, Gbenga Habeeb Olufemi

**Affiliations:** 1 Department of Anaesthesia, University of Ilorin Teaching Hospital, Ilorin, Kwara State; 2 Department of Surgery, University of Ilorin Teaching Hospital, Ilorin

**Keywords:** Knowledge, practice, healthcare workers, hospital acquired infections, intensive care unit, infection control

## Abstract

**Background:**

Hospital-acquired infections (HAIs), one of the leading causes of, morbidity and mortality, are common in developing countries. Methicillin-resistant *staphylococcus aureus* (MRSA), commonest cause of HAIs, has been isolated from the hands of more than half of health care workers. Practice of hand hygiene may help in the control of nosocomial infections. We evaluated the practice of infection control among health care workers in the intensive care unit (ICU) of our hospital.

**Materials and methods:**

This is a descriptive cross-sectional study. Information on knowledge, awareness and practice of infection control in the ICU were obtained from health care workers with the aid of a structured questionnaire.

**Results:**

Sixty nine out of the 80 (86%) respondents had good knowledge that a hand is the most common vehicle of transmission of infection. However, 53.8% and 32.5% of the respondents had knowledge of movement of hand hygiene and practiced six steps of the hand washing technique respectively. Though, physicians accounted for 68 (85%) of the respondents, only 28% of them practiced the six steps of the hand washing technique with resident doctors constituting a large proportion of hand washing technique defaulters. Only 13.9% of non-physician and 7.5% of physician respondents had ever attended a training program on infection control respectively

**Conclusion:**

Knowledge and awareness of infection control among the health care workers in our ICU is good but the practice is poor. Training workshop on infection control should be organized for all ICU health care workers to reduce noso-comial infections.

## Introduction

Infection is a major problem encountered in health care delivery services worldwide. It constitutes one of the most important causes of morbidity and mortality associated with clinical, diagnostic and therapeutic procedures.^1^,^2^ Hospital acquired infections are a major setback in the care of patients as a result of increased healthcare cost and over stretching of the available heath care resources. Nosocomial or hospital acquired infection (HAI) is a serious public health issue;^3^ about 1.4 million people across the world are infected at any given time. It has been estimated that almost 10% of all hospitalized patients would develop one form of infection or the other during the course of their stay in the hospital.^4^,^5^ The menace of condition is bothersome due to its high prevalence rate of 30–50% in developing countries.^6^,^7^ HAI result in substantial morbidity and are estimated to cause about 80,000 deaths annually in the United State.^8^ Koch et al reported a higher risk of death within 30 days in patients with HAI and 1 year when compared to those without HAIs.^9^ The most important source of spread of these infections is through the contaminated hands of the health care givers: doctors, nurses and other staffs.^7^ Most hospital acquired infections are caused by transmission of pathogens from one patient to another, especially by health care workers who failed to wash their hands after evaluating a patient, or who did not properly comply with simple hospital hygiene measures.^10^ HAI vary according to the type of clinical department, with the highest infection rate usually found in the intensive care units, neonatal and burns unit^9^. Therefore the propensity for developing HAIs is higher in critically ill patients admitted to intensive care unit (ICU). The presence of multiple invasive devices for treating or monitoring the care of such patients makes them vulnerable to common noso-comial infections like urinary tract infection( UTI), respiratory tract infection( RTI) etc. Fadeyi et al^11^ showed that 17% of patients under critical care had MRSA bacteraemia, and MRSA carrier status of 52.5% among health care workers in the critical care unit with doctors and nurses accounting for 22.7% and 16.7% respectively. Adherence to the guidelines on infection control by ICU health care givers is encouraged in order to minimize the incidence of cross-infection of a patient to another with virulent organism during care.

A study showed that practice of hand hygiene among health care workers is generally low.^12^ Furthermore, several studies done to assess the knowledge, attitudes and compliance and reasons for non-adherence to hand hygiene guidelines revealed that compliance with hygiene protocols by health care workers is poor^13^,^14^,^15^ due to several constraints, including heavy work load, high number of clinical procedures and skin conditions of health care worker.^16^,^17^ It is alarming that compliance with general aseptic guidelines was found to be worse before high risk procedures were done.^14^,^18^ Thus, the reported low or outright non-compliance of health care workers with the global best practices on the prevention of spread of hospital acquired infections and its associated morbidity and mortality makes this study a necessity. Therefore, the aim of the study was to assess the knowledge, awareness and practice of infection control, among hospital staff in the ICU of our hospital.

## Materials and methods

After Institutional Ethical Committee approval, this prospective cross-sectional study was conducted in our 3 bedded ICU from August 2016 to October 2016. It was a questionnaire-based survey and the 15- item questionnaires were distributed to all consenting health care worker involved in the care of patients in ICU during the study period. The respondents were urged to fill the questionnaires and return them on the same day to avoid bias and collaboration amongst them.

The questionnaire covered the demographic variables of the health care workers. It also assessed their knowledge, awareness and practice of infection control in the ICU based on universal protocol.

The data was analyzed using Statistical Package for Social Sciences (SPSS software version 20). Results were expressed in means and percentages, represented in tables. All statistical tests were deemed significant at p value of < 0.05.

## Results

### Demography

Eighty five questionnaires were sent out and eighty questionnaires were returned fully completed; and were analyzed. The respondents included physicians 68 (85%) and non-physicians 12 (15%). Of the 68 physicians that participated in the survey, consultants (C) were 10 (12.5%), senior residents (SR) were 27 (33.8%), junior residents (JR) were 22 (27.5%), and Interns (I) were 9 (11.3%). Of the 12 non-physicians respondents, nurses (N) and physiotherapists were 8 (10%) and 4 (5%) respectively, Table I.

**Table 1:** Distribution of health personnel

	Frequency (N)	Percentage (%)
Consultant	10	12.5
Senior Registrar	27	33.8
Registrar	22	27.5
House Officer	9	11.3
Nurse	8	10.0
Physiotherapist	4	5.0
Total	80	100

### Knowledge

Sixty nine out of 80 (86.3%) respondents acknowledged that the hand was the most common vehicle of transmission of infection among patients in the intensive care unit (ICU). Though physicians accounted for 85% of the respondents, 61 (90%) of them demonstrated knowledge that the hand is the vehicle of transmission. Of the non-physicians respondents involved in the management of ICU patients, 4 (50%) of the 8 nurses and all the 4 (100%) physiotherapists showed similar knowledge that the hand was the vehicle of transmission. Overall, 43 out of 80 (53.8%) respondents had good knowledge of 5 movements of hand hygiene. However, the proportion of respondents with poor knowledge of 5 movement of hand hygiene was higher among residents: 15 (55.5%), 13 (59.1%) and 5 (55.5%) of the senior registrars (SR), registrars (R) and interns (I) respectively.

Though 42 out of the 80 (52.5%) respondents had knowledge regarding the six stages of hand washing, 77 out of the 80 (96.3%) of the respondents agreed that good knowledge of hand washing is an indispensible part of a hygienic culture in the ICU.

### Practice

The practice of hand hygiene which includes the five movements of hand hygiene and the six stages of hand washing by the respondents involved in the care of ICU patients was poor as only 26 out of the 80 respondents, (32.5%) practiced the hand hygiene respectively. However, it was noted that greater percentage of non- physicians, 7 out of 12 (58.3%) followed the six stages of hand washing compared with the physicians, 19 out of 68 (27.9%) and the difference was statistically significant p< 0.038.

Majority of the respondents, 70 out of 80 (87.5%) indicated that there was a designated area for hand washing in the ICU. However, 52 out of 80 (65%) of respondents admitted that there was no running water at the designated area. Majority of the respondents, 74 of the 80 respondents (92.5%), used soap and water provided at the designated area. Similarly, the practice of wearing fresh gloves before examining new patients was only adhered to by 41of the 80 (51.2%) respondents. Poor adherence to the practice was observed among residents; 50%, 40.7% and 22.2% for the registrars, senior registrars and interns respectively. Twenty six of the 80 (32.5%) respondents routinely masked and gowned before entering the intensive care unit and this was poor. Surprisingly, majority of the non-physicians (75%) and a quarter of physicians (25%) had good attitudes towards masking and gowning and this was significant statistically, p=0.001. Majority of the respondents (93.7%) said the supplied gowns were non-disposable.

On the infection control protocol, 18 out of 80 of the respondents (22.5%) said that they are aware of guidelines/protocols for infection control, 73.7% were not aware and 3.7% did not know about its existence. However, only 17 out of the 80 respondents (21.2%) admitted to following the guidelines.

Majority of the respondents (70%) believed that the quality of sanitation in the ICU was good, while 3.8% and 26.2% of them believed scored the quality of the ICU as excellent and bad respectively.

The aseptic technique approach in the care of patients, in terms of sterility of the equipment and consumables used in the ICU, was adjudged as properly or poorly sterilized by 49 out of the 80 (60.7%) and 31 out of the 80 (39.3%) of the respondents respectively.

Also, 13 out of the 80 (16.3%) respondents had knowledge of the existence of universal guidelines/protocol for catheter care in ICU However, 77 out of the 80 (96.2%) respondents adhered to good catheter care and believed it helps in the control of infection in patients.

### Awareness

Most of the respondents 78 out of the 80 (98.7%) agreed that training programs and awareness campaigns would be helpful in preventing infections in the ICU. However, only 6 out of the 12 non-physician (50%) respondents and 5 out of 68 (7.3%) physician respondents had attended any training program on infection control.

## Discussion

This study showed that health care workers had a good knowledge (86.3%) that the hand was the most common vehicle of transmission of infection. It also revealed that 53.8% of the health workers in our ICU had knowledge about the five movements of hand washing and 52.8% about the six steps of hand washing. The actual compliance practices with the five movements of hand washing and for six steps of hand washing were 32.6% and 33.5% respectively, which rather lower compared with results of other similar published work^19^. A meta-analysis of the compliance of health personnel towards hand hygiene, specifically hand washing, was 52% with a range of 27–86%.^19^ Infection control practices are of paramount importance in the intensive care unit (ICU). Hand hygiene is the initial step towards successful infection control in any health care set up.^20^ We also found that residents and interns had moderate knowledge about hand hygiene and this is similar to the reported outcome of similar work conducted by Colosi et al.^21^ The poor compliance of residents with hand hygiene in our study, compared with other health care workers, is comparable with findings from similar studies done in Iran, Kuwait and India.^22^,^23^ This poor compliance, in addition to inadequate knowledge, may also be due to lack of proper facilities such as uninterrupted running tap, soap, gowns and masks among others necessary for prevention of transmission of infections in the ICU.

Provision of colour-coded dustbin/bags that ensure proper disposal of biomedical wastes such as needles, syringes, used gloves also contribute to low rate of transmission of infections among ICU patients.^24^ Despite the fact that colour-coded dustbins were supplied to our ICU, about 34 (42.5%).of respondents admitted to disposing used biomedical wastes into such dustbins. This displayed ignorance is indicative of lack of education/training on infection control.

Previous observational studies have found that nurses had a better practice of hand hygiene than doctors.^25^,^26^ Results of the present study is in agreement with the finding especially when compared with the resident doctors.

The guidelines of hand hygiene are simple and easy to learn, however adhering to the guidelines in a working environment seems to be a challenge. A study carried out in a Nigerian tertiary institution showed that out of the 198 health workers screened for carrier status of Methicilin Resistant *Staphyloccous arueus* (MRSA), 104 healthcare workers in the critical units had MRSA with nasal carriage accounting for 38.9% and hand carriage 25.3%.^11^ Doctors and nurses were found to be the predominant carriers of MRSA^10^ 22.7% and 16.7% respectively. In the present study, only 51.3% always wore new gloves before touching a new patient and this poor compliance with guidelines on hand hygiene by health care workers involved in the care of ICU could predispose to healthcare associated infections. This can negatively impact on patients, visitors and health care workers and could probably have accounted for the high level of MRSA amongst the doctors and nurses observed in the study of Fadeyi et al.^11^

This present study showed that only 11 (13.9%) of the health workers had ever attended any form of training program on infection control and 5 (7.3%) of the physicians involved in the care of critically ill patients had attended training. This is worrisome due to the possibility of high rate of development of hospital acquired infections by patients in our ICU. Studies have proved that training of health care workers on infection control has a beneficial impact on all medical staff by improving their compliance with the hand hygiene guidelines.^27^ Since training builds capacity, which has a positive association with compliance with infection control measures there is an urgent need to organize a workshop on infection control for health care workers in our facility in order to maintain high quality infection control practices. The workshops should be organized on regular basis and this should be complemented by adequate hygiene supervision of health care personnel in the hospital. This would help health workers understand the importance of infection control and hand hygiene in reducing transmission of infection in the Intensive care unit.

Limitations of our study included the small number of respondents and restriction of the survey to health care workers in our ICU.

## Conclusion

Despite the good knowledge and awareness of universal protocol for the prevention of nosocomial infections in ICU, the adherence to the protocol is poor among the health workers surveyed in our facility. It therefore recommended that the institution should have regular educational programs on infection control for all health care workers involved in the care of patients in the intensive care unit. The institution should have a clearly written infection control guideline placed in strategic position in the ICU. There should be a regular system of monitoring infection rates as well as disseminating information which will serve as a link between the management and the health care workers.

## Figures and Tables

**Table 2:** Knowledge of study group regarding hand washing

	No (%) of consultant giving correct response	No (%) of senior registrars giving correct response	No of registrars giving correct response	No (%) of interns giving correct response	No (%) of nurses giving correct response	No (%) of physiotherapists giving correct response
Knowledge of five movements of hand hygiene	9 (90)	12 (44)	9 (40)	4 (44)	5 (62)	4 (100)
Hand the most common vehicle of infection transmission	8 (80)	24 (88)	20 (90)	9 (100)	4 (50)	4 (100)
Knowledge of six stages of hand washing	8 (80)	15 (55)	8 (36)	3 (33)	5 (62)	3 (75)
Hand washing should become an indispensible part of hygienic culture in ICU	10 (100)	26 (96)	22(100)	7 (77)	8 (100)	4 (100)
